# Modeling of the aorta artery aneurysms and renal artery stenosis using cardiovascular electronic system

**DOI:** 10.1186/1475-925X-6-22

**Published:** 2007-06-09

**Authors:** Kamran Hassani, Mahdi Navidbakhsh, Mostafa Rostami

**Affiliations:** 1 Biomedical Engineering Department, Amirkabir University, Tehran, Iran; 2 Mechanical Department, Iran University of Science and Technology, Tehran, Iran; 3 Biomedical Engineering Department, Amirkabir University, Tehran, Iran

## Abstract

**Background:**

The aortic aneurysm is a dilatation of the aortic wall which occurs in the saccular and fusiform types. The aortic aneurysms can rupture, if left untreated. The renal stenosis occurs when the flow of blood from the arteries leading to the kidneys is constricted by atherosclerotic plaque. This narrowing may lead to the renal failure. Previous works have shown that, modelling is a useful tool for understanding of cardiovascular system functioning and pathophysiology of the system. The present study is concerned with the modelling of aortic aneurysms and renal artery stenosis using the cardiovascular electronic system.

**Methods:**

The geometrical models of the aortic aneurysms and renal artery stenosis, with different rates, were constructed based on the original anatomical data. The pressure drop of each section due to the aneurysms or stenosis was computed by means of computational fluid dynamics method. The compliance of each section with the aneurysms or stenosis is also calculated using the mathematical method. An electrical system representing the cardiovascular circulation was used to study the effects of these pressure drops and the compliance variations on this system.

**Results:**

The results showed the decreasing of pressure along the aorta and renal arteries lengths, due to the aneurysms and stenosis, at the peak systole. The mathematical method demonstrated that compliances of the aorta sections and renal increased with the expansion rate of the aneurysms and stenosis. The results of the modelling, such as electrical pressure graphs, exhibited the features of the pathologies such as hypertension and were compared with the relevant experimental data.

**Conclusion:**

We conclude from the study that the aortic aneurysms as well as renal artery stenosis may be the most important determinant of the arteries rupture and failure. Furthermore, these pathologies play important rules in increase of the cardiovascular pulse pressure which leads to the hypertension.

## Background

The aortic aneurysm is a disease, which is defined as focal or diffuse dilatation of the aorta. Most of the aorta aneurysms are fusiform (concentric radial dilatation) but infrequently may be saccular (eccentric radial dilatation). The abdominal aorta aneurysm is the most common form of the aneurismal disease. Less commonly patients may present with the thoracic aorta aneurysms. The aorta aneurysms have primarily been studied by Long [1] and Morris [2] who investigated the compliance of aortic aneurysms and observed the effects of the pathology. More recent studies have used the clinical data to investigate the aortic aneurysms [3-5].

On the other hand, the cardiovascular disease is very common in the patients with the renal artery stenosis. The two main causes of the renal artery stenosis are atherosclerosis and fibro muscular disease. Woolfson [6], Bude [7], Nawaz khan [8] and Coen [9] have shown that the renal artery stenosis may lead to the hypertension, fluid retention, progressive renal failure and flash pulmonary edema. In this study, we have taken a slightly different approach to the modelling of the cardiovascular disorders and tried to exhibit the effects of the aortic aneurysms and renal artery stenosis on the cardiovascular system using the combination of the haemodynamic and electrical parameters. The principal goal of this study is to present the possibility of modelling the cardiovascular pathologies, such as the aortic aneurysms or renal artery stenosis, using an electronic circuit representing the whole cardiovascular system. To realize this aim, this study includes the following four components:

1) A brief review of the electronic cardiovascular system which has been previously described in detail [10].

2) The computational fluid dynamics method which has been used to compute the pressure drops due to the pathologies.

3) The mathematical method which has been used to calculate the variations of the compliance due to the pathologies.

4) The study of the effects of the pathologies using the electronic cardiovascular system and investigating the cases.

The obtained results have been compared with the relevant clinical data to determine whether our modelling method has been able to present the effects of the pathologies appropriately.

## Methods

### The electronic cardiovascular system review

**Figure 1:**
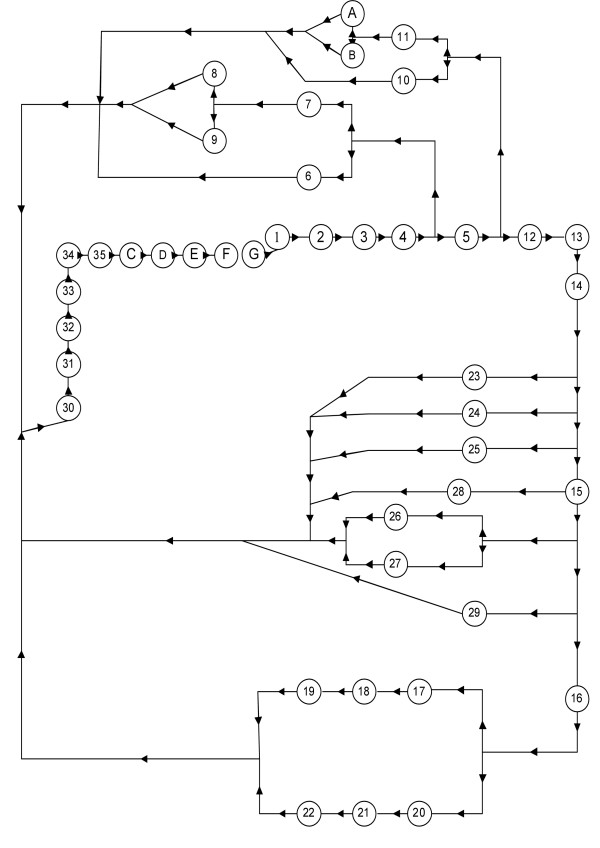
**The block diagram and specifications of the system's elements**. The block diagram of the cardiovascular system.

**Figure 2:**
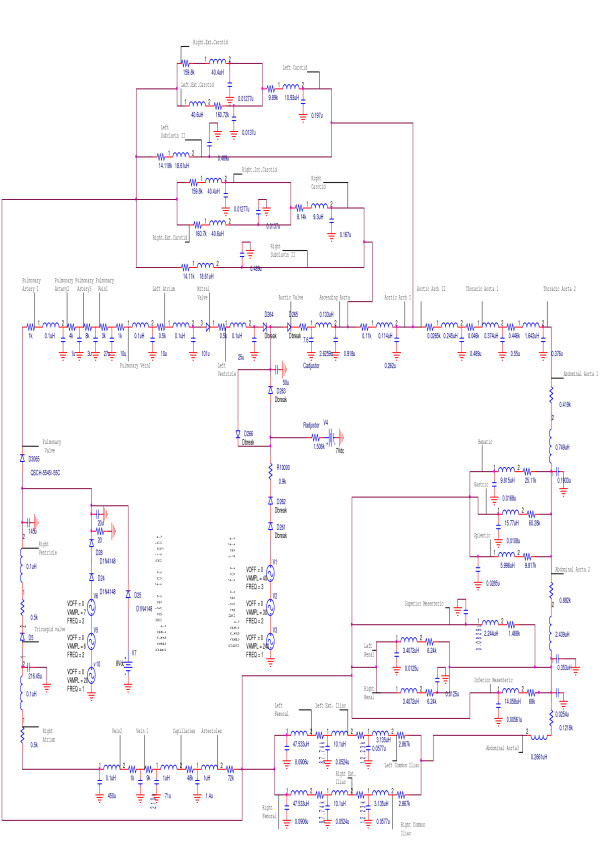
**Electrical circuit of the cardiovascular system**. The electrical circuit of the cardiovascular system including 42 elements. Each element is named on the circuit.

The lumped parameter model of the cardiovascular system including the block diagram and the specifications of each element is presented in Figure 1 and Table 1. The equivalent model of the system including the pulsatile heart and the arterial tree is illustrated in Figure 2 in terms of its electrical circuit analog, in which voltage(volt) is analogous to pressure(1 mmHg), capacitance(1000 μF), to compliance(1 ml/Pa), resistance(1 kΩ), to resistance(1 Pa.s/ml)and inductance(1 μH) to inertance(1 Pa.s^2^/ml). The electrical model consists of forty two elements which represent the left and right ventricles, systemic arteries and veins, and pulmonary arteries and veins. Each element consists of a conduit for viscous blood flow, which is characterized by a linear resistance and a volume storage element, which is characterized by a linear capacitor. The inertance of each element is characterized by a linear inducer. The reference pressure is atmosphere pressure (or ground) for the circuit. The energy of systolic contraction is modeled by superposition of three voltage suppliers and two ideal diodes. The voltage suppliers vary periodically over time and are responsible for driving the flow of blood in both left and right ventricles. The four ideal diodes represent the ventricular inflow and outflow valves and ensure unidirectional blood flow; furthermore the model works at the frequency of 1 Hz. We have demonstrated [10] that this model behaves reasonably in terms of pulsatile waveforms and properties of the systemic circulation.

**Table 1:** The electrical specifications of each element. The electrical characteristics of the elements of the system with reference to the block diagram of Figure 1.

ITEM	DESCRIPTION	R(kΩ)	L(μH)	C(μF)
1	Left Atrium	0.5	0.1	101
2	Left Ventricle	0.5	0.1	25
3	Ascending Aorta	7.6	0.133	0.918
4	Aortic Arch 1	0.011	0.144	0.262
5	Aortic Arch 2	0.0265	0.245	0.489
6	Right Subclavin II	14.118	18.61	0.489
7	Right Carotid	8.14	9.3	0.167
8	Right int Carotid	159.8	40.4	0.01277
9	Right ext Carotid	160.7	40.6	0.0137
10	Left Subclavin II	14.118	18.61	0.489
11	Left Carotid	9.89	10.93	0.197
A	Left int Carotid	159.8	40.4	0.01277
B	Left ext Carotid	160.72	40.6	0.0137
12	Thoracic Aorta 1	0.046	0.374	0.556
13	Thoracic Aorta 2	0.446	1.642	0.376
14	Abdominal Aorta 1	0.419	0.749	0.1933
15	Abdominal Aorta 2	0.882	2.439	0.353
16	Abdominal Aorta 3	0.1218	0.2661	0.0254
17	Left Common Iliac	2.867	3.135	0.0557
18	Left External Iliac	12.23	10.1	0.0524
19	Left Femoral	87.74	47.533	0.0906
20	Right Common Iliac	2.867	3.135	0.0577
21	Right External Iliac	12.23	10.1	0.0524
22	Right Femoral	87.74	47.533	0.0906
23	Hepatic	25.11	9.815	0.0168
24	Oastric	60.28	15.77	0.0108
25	Splenic	9.817	5.996	0.0285
26	Left Renal	6.24	3.4072	0.0125
27	Right Renal	6.24	3.4072	0.0125
28	Superior Mesenteric	1.468	2.244	0.0829
29	Inferior Mesenteric	68	14.058	0.00561
30	Arterioles	72	1	1.4
31	Capillaries	48	1	71
32	Vein 1	9	-	210
33	Vein 2	1	0.1	450
34	Right Atrium	0.5	0.1	216.45
35	Right Ventricle	0.5	0.1	150
C	Pulmonary Artery 1	1	0.1	1
D	Pulmonary Artery 2	4	-	1
E	Pulmonary Artery 3	8	-	3
F	Pulmonary Vein 1	3	-	27
G	Pulmonary Vein 2	1	0.1	10

### The applications of computational fluid dynamics method (CFD) to the modelling

#### 1) The aorta aneurysms

**Figure 3:**
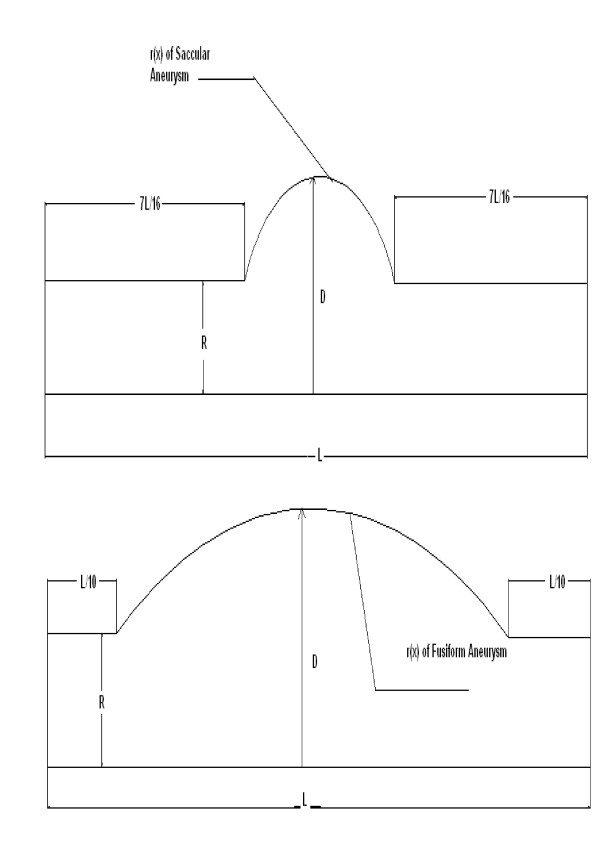
**The geometrical model of the aortic aneurysms**. The geometry of aortic saccular aneurysm(above) and the geometry of fusiform aneurysm(under). Both aneurysms are located in the center of the artery.

The geometrical model of each aorta section with the aneurysm was made according to the anatomical data [11] and illustrated in Figure 3. The locations of the hypothetical aneurysms including fusiform and saccular, were placed in the middle of the studied sections. In our other study [12] including 100 patients, we observed that the saccular aneurysms occur mostly in the abdominal aorta and rarely in the thoracic aorta where the fusiform aneurysms occur both in the abdominal and thoracic aorta sections. The geometry of the saccular aneurysm is assumed by

*r*(*x*) = *ax*^2 ^+ *bx *+ *c*

The geometry of the fusiform aneurysm is assumed by

*r*(*x*) = *a *sin *π*.*x *+ *b*

The a, b and c coefficients can be easily calculated by the geometrical model of each aorta section, considering the different aneurysm expansion rates, according to Figure 3. In this geometry, (R) is the tube radius of the unobstructed part, (L) is the total length of the tube and (D) is the value of the maximum posterior displacement of the aneurismal wall which is determined by expanding the tube radius (R) with the following rate

*D *= *R *+ *n*

The parameter (n) is 20%R, 40%R, 50%R, 70%R and 90%R representing the aneurysm expansion rate. It has been assumed that blood flow is represented by a laminar, incompressible, ideal and Newtonian fluid of constant viscosity, and density. The appropriate experimental equation for blood flow subject to the aorta artery has been expressed by Womersley [13] as a sinusoidal function, with frequency of 1 Hz,

*Q = 1.05+2.407 Sin (6.283t+0.552)+2.324 Sin (12.566t-1.096) -0.921 Sin (18.849t+0.7384) -0.398 Sin (25.132t-0.082)*

The parameter (Q) is the volumetric flow rate (ml/s). The velocity (U) at any given cross section of the aorta artery with radius (R) can be written as

U=Qπ.R2
 MathType@MTEF@5@5@+=feaafiart1ev1aaatCvAUfKttLearuWrP9MDH5MBPbIqV92AaeXatLxBI9gBaebbnrfifHhDYfgasaacH8akY=wiFfYdH8Gipec8Eeeu0xXdbba9frFj0=OqFfea0dXdd9vqai=hGuQ8kuc9pgc9s8qqaq=dirpe0xb9q8qiLsFr0=vr0=vr0dc8meaabaqaciaacaGaaeqabaqabeGadaaakeaacqWGvbqvcqGH9aqpdaWcaaqaaiabdgfarbqaaGGaciab=b8aWjabc6caUiabdkfasnaaCaaaleqabaacbaGae4Nmaidaaaaaaaa@3513@

Gambit software can automatically generate a mesh for any geometry. The uniform meshes were used for each of the aorta sections with the aneurysms. The quadrilateral and mappable elements were used for the fluid domain. We have used the computational fluid dynamics method, fluent code, to compute the pressure drops of the sections with the aneurysms. The velocity waveform, expressed by equation (5) was applied at the inlet boundary of each studied section. Blood properties chosen were viscosity of 0.0035 (Pa.s) and density of 1050 (kg/m^3^). The outlet was assumed as the zero pressure state and the inlet pressure was computed by the code. The solution method was 2D, unsteady, 1^st^-order implicit, segregated and axisymmetric. The time step size was 0.1 (s) and typically 10–20 iterations were required per time steps. The simulation reached nearly steady state oscillation after the second cycle. The pressure difference between the inside and outside of each aorta section was recorded.

#### 2) The renal artery stenosis

The geometrical model of the renal artery with the stenosis was made using the anatomical data [11] and illustrated in Figure 3. The renal artery stenosis due to arterial dysplasia generally affects the middle and distal renal artery in the patients [8], therefore we located the hypothetical stenosis in the middle of the artery. The geometry of the renal artery stenosis is assumed by

*r*(*x*) = *ax*^2 ^+ *bx *+ *c*

**Figure 4:**
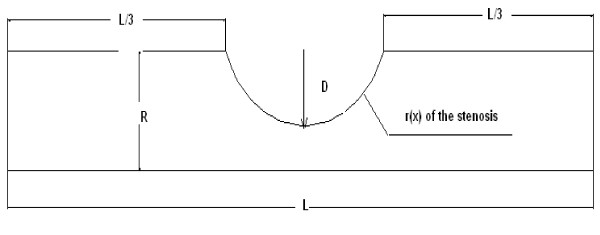
**The geometrical model of the renal stenosis**. The geometry of renal stenosis which is located in the centre.

The a, b and c coefficients were calculated by geometrical model of the artery according to Figure 4. In this geometry, (R) is the tube radius of the normal part; (L) is the total length of the tube and (D) is the value of the maximum interior displacement of the wall with the stenosis and determined by narrowing the tube radius (R) with the following rate

*D *= *R *- *n*

The parameter (n) is 20%R, 40%R, 50%R, 70%R and 90%R representing the stenosis narrowing rate. The peak systolic velocity in the renal artery averages 120 ± 12 cm/s) [8]. We assumed a simple sinusoidal function for blood velocity subject to the renal artery which is given by

*V *= *1.2 Sin(6.283t+0.552)*

The parameter (V) is according to (m/s) unit and the frequency of the equation is 1 Hz. The pressure difference between the inside and the outside of the renal artery with different stenosis rates was computed and recorded using the same method which was described above in section A.

### The applications of the mathematical method to the modelling

The compliances of the aortic aneurysms and the renal artery stenosis were computed by the mathematical method. The compliance of an elastic vessel is defined by [14]

C=3π.R3.Z2Eh
 MathType@MTEF@5@5@+=feaafiart1ev1aaatCvAUfKttLearuWrP9MDH5MBPbIqV92AaeXatLxBI9gBaebbnrfifHhDYfgasaacH8akY=wiFfYdH8Gipec8Eeeu0xXdbba9frFj0=OqFfea0dXdd9vqai=hGuQ8kuc9pgc9s8qqaq=dirpe0xb9q8qiLsFr0=vr0=vr0dc8meaabaqaciaacaGaaeqabaqabeGadaaakeaacqWGdbWqcqGH9aqpdaWcaaqaaiabiodaZGGaciab=b8aWjabc6caUiabdkfasnaaCaaaleqabaGaeG4mamdaaOGaeiOla4IaemOwaOfabaGaeGOmaiJaemyrauKaemiAaGgaaaaa@3A44@

The parameter (R) is the radius of the vessel, (Z) is the length of the vessel, (h) is the thickness of the vessel, and (E) is the elastic module of the vessel. The normal compliances of the aorta artery sections and the renal artery were computed by equation (9). In order to calculate the saccular aneurysms, we substituted equation (1) into equation (9) to give

dC=3π.(ax2+bx+c)3dx2Eh
 MathType@MTEF@5@5@+=feaafiart1ev1aaatCvAUfKttLearuWrP9MDH5MBPbIqV92AaeXatLxBI9gBaebbnrfifHhDYfgasaacH8akY=wiFfYdH8Gipec8Eeeu0xXdbba9frFj0=OqFfea0dXdd9vqai=hGuQ8kuc9pgc9s8qqaq=dirpe0xb9q8qiLsFr0=vr0=vr0dc8meaabaqaciaacaGaaeqabaqabeGadaaakeaacqWGKbazcqWGdbWqcqGH9aqpdaWcaaqaaiabiodaZGGaciab=b8aWjabc6caUiabcIcaOiabdggaHjabdIha4naaCaaaleqabaGaeGOmaidaaOGaey4kaSIaemOyaiMaemiEaGNaey4kaSIaem4yamMaeiykaKYaaWbaaSqabeaacqaIZaWmaaGccqWGKbazcqWG4baEaeaacqaIYaGmcqWGfbqrcqWGObaAaaaaaa@4689@

Integrating equation (10) using the geometry of Figure 2 and the condition X_0 _= 7L/16, X_1 _= 9L/16, we have

Csaccularaneurysm=∫X0X13π.(ax2+bx+c)3dx2Eh
 MathType@MTEF@5@5@+=feaafiart1ev1aaatCvAUfKttLearuWrP9MDH5MBPbIqV92AaeXatLxBI9gBaebbnrfifHhDYfgasaacH8akY=wiFfYdH8Gipec8Eeeu0xXdbba9frFj0=OqFfea0dXdd9vqai=hGuQ8kuc9pgc9s8qqaq=dirpe0xb9q8qiLsFr0=vr0=vr0dc8meaabaqaciaacaGaaeqabaqabeGadaaakeaaieWacqWFdbWqdaWgaaWcbaGae83CamNae8xyaeMae83yamMae83yamMae8xDauNae8hBaWMae8xyaeMae8NCaiNae8hiaaIae8xyaeMae8NBa4Mae8xzauMae8xDauNae8NCaiNae8xEaKNae83CamNae8xBa0gabeaakiabg2da9maapedabaWaaSaaaeaacqaIZaWmiiGacqGFapaCcqGGUaGlcqGGOaakcqWGHbqycqWG4baEdaahaaWcbeqaaiabikdaYaaakiabgUcaRiabdkgaIjabdIha4jabgUcaRiabdogaJjabcMcaPmaaCaaaleqabaGaeG4mamdaaOGaemizaqMaemiEaGhabaGaeGOmaiJaemyrauKaemiAaGgaaaWcbaGaemiwaGLaeGimaadabaGaemiwaGLaeGymaedaniabgUIiYdaaaa@6287@

We performed the similar mathematical method for the fusiform aneurysms and got

CFusiformaneurysm=∫X0X13π.(asin⁡π.x+b)3dx2Eh
 MathType@MTEF@5@5@+=feaafiart1ev1aaatCvAUfKttLearuWrP9MDH5MBPbIqV92AaeXatLxBI9gBaebbnrfifHhDYfgasaacH8akY=wiFfYdH8Gipec8Eeeu0xXdbba9frFj0=OqFfea0dXdd9vqai=hGuQ8kuc9pgc9s8qqaq=dirpe0xb9q8qiLsFr0=vr0=vr0dc8meaabaqaciaacaGaaeqabaqabeGadaaakeaaieWacqWFdbWqdaWgaaWcbaGae8NrayKae8xDauNae83CamNae8xAaKMae8NzayMae83Ba8Mae8NCaiNae8xBa0Mae8hiaaIae8xyaeMae8NBa4Mae8xzauMae8xDauNae8NCaiNae8xEaKNae83CamNae8xBa0gabeaakiabg2da9maapedabaWaaSaaaeaacqaIZaWmiiGacqGFapaCcqGGUaGlcqGGOaakcqWGHbqycyGGZbWCcqGGPbqAcqGGUbGBcqGFapaCcqGGUaGlcqWG4baEcqGHRaWkcqWGIbGycqGGPaqkdaahaaWcbeqaaiabiodaZaaakiabdsgaKjabdIha4bqaaiabikdaYiabdweafjabdIgaObaaaSqaaiabdIfayjabicdaWaqaaiabdIfayjabigdaXaqdcqGHRiI8aaaa@6477@

Where X_0 _= L/10 and X_1 _= 2L/3, according to the geometry in Figure 3. The total compliance of each aorta artery section with the saccular or fusiform aneurysms is given by

***C***_***total ***_= ***C***_***normal parts ***_+ ***C***_***aneurysmal parts***_

Again, the compliance of the unobstructed parts was calculated using equation (9). The compliance of the renal stenosis is determined with the same mathematical method and given by

Crenalstenosis=∫X0X13π.(ax2+bx+c)3dx2Eh
 MathType@MTEF@5@5@+=feaafiart1ev1aaatCvAUfKttLearuWrP9MDH5MBPbIqV92AaeXatLxBI9gBaebbnrfifHhDYfgasaacH8akY=wiFfYdH8Gipec8Eeeu0xXdbba9frFj0=OqFfea0dXdd9vqai=hGuQ8kuc9pgc9s8qqaq=dirpe0xb9q8qiLsFr0=vr0=vr0dc8meaabaqaciaacaGaaeqabaqabeGadaaakeaaieWacqWFdbWqdaWgaaWcbaGae8NCaiNae8xzauMae8NBa4Mae8xyaeMae8hBaWMae8hiaaIae83CamNae8hDaqNae8xzauMae8NBa4Mae83Ba8Mae83CamNae8xAaKMae83Camhabeaakiabg2da9maapedabaWaaSaaaeaacqaIZaWmiiGacqGFapaCcqGGUaGlcqGGOaakcqWGHbqycqWG4baEdaahaaWcbeqaaiabikdaYaaakiabgUcaRiabdkgaIjabdIha4jabgUcaRiabdogaJjabcMcaPmaaCaaaleqabaGaeG4mamdaaOGaemizaqMaemiEaGhabaGaeGOmaiJaemyrauKaemiAaGgaaaWcbaGaemiwaGLaeGimaadabaGaemiwaGLaeGymaedaniabgUIiYdaaaa@5E88@

Where X_0 _= L/3 and X_1 _= 2L/3 in accordance with the geometry in Figure 4. The total compliance of the renal artery with the stenosis is also given by

***C***_***total ***_= ***C***_***normal parts ***_+ ***C***_***renal stenosis***_

### The applications of the cardiovascular electronic system to the modelling

The electronic circuit of the aorta artery, shown in Figure 2, consists of six resistors, capacitors and inducers. The computed pressure drop and the compliance of each aorta section with the aneurysms were converted to their electrical counterparts including resistance and capacitance. These new values of the resistances and the capacitances were applied to the relating part on the aorta circuit. The whole cardiovascular electronic system was run to the time, 100 s, and the results were obtained and recorded. The similar method was used to study the effects of the renal stenosis on the electronic cardiovascular system.

## Results

### 1) The aorta artery aneurysms

**Figure 5:**
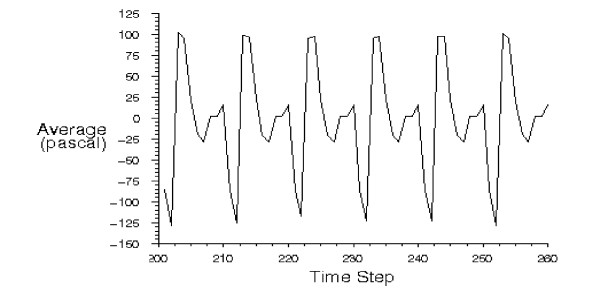
**The pressure graph of the abdominal III aorta with 90% aneurysm**. The inlet pressure waive form of abdominal III aorta with 90% expansion rate of saccular aneurysm obtained by CFD method.

**Figure 6:**
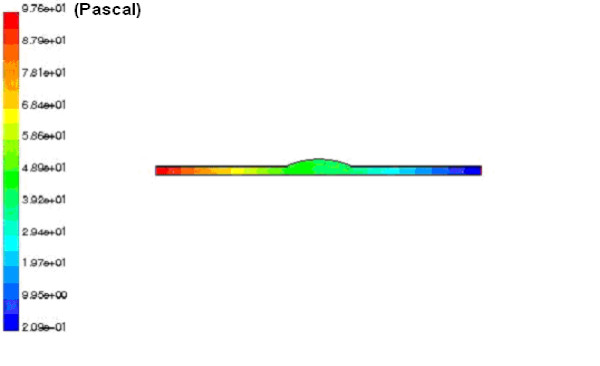
**The pressure contour of the abdominal III aorta with 90% aneurysm**. The pressure contour of abdominal III aorta with 90% saccular aneurysm obtained by CFD method. The pressure(pascal) decreases along the artery length.

**Figure 7:**
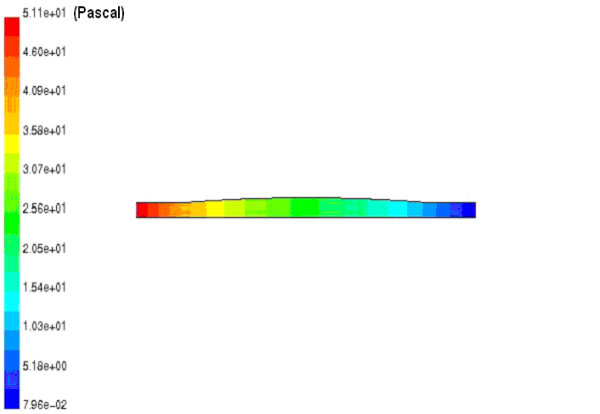
**The pressure contour of the thoracic I aorta with 50% aneurysm**. The pressure contour of abdominal III aorta with 90% fusiform aneurysm obtained by CFD method. The contour shows the variations of the pressure(pascal) along the artery.

**Figure 8:**
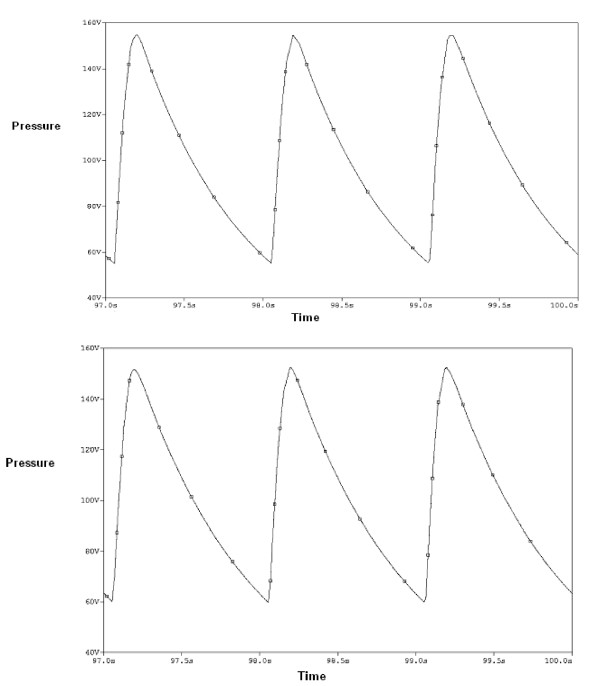
**The electrcal pressure graphs of the abdominal I aorta with 20% saccular aneurysm(above) and abdominal II aorta with 40% fusiform aneurysm(under)**. The pressure waive forms of the abdominal I aorta with the 20% saccular aneurysm(above) which obtained from the electrical circuit as well as abdominal II aorta with the 40% fusiform aneurysm(under).

Table 2 shows the results of the modeling including the pressure drops, at peak systole, and the compliances of different aorta sections with the aneurysms. The pressure wave form at the inlet of the abdominal III aorta, with 90% expansion rate of the saccular aneurysm, is presented in Figure 5. We see the pressure rises above zero (97.6 Pa) at the peak systole and reaches a minimum value (-110 Pa) at the max reverse flow. A negative pressure shows the decelerative phase of the aortic flow. The pressure contour of this section is also presented in Figure 6. The pressure decreases along the artery length at the peak systole. The similar pressure contour is presented for thoracic I aorta with 50% expansion rate of the fusiform aneurysm in Figure 7. We studied the effects of the aorta aneurysms on the electronic circuit of the cardiovascular system as well. Figure 8 presents the diastole/systole pressure graph of the abdominal I aorta with 20% expansion rate of the saccular aneurysm. The pressure waveform varies between 55 mmHg (volt) to 155 representing diastolic and systolic pressure values. This figure also shows the variation of the pressure during the cardiac cycle for the abdominal II aorta with 40% expansion rate of the fusiform aneurysm.

**Table 2:** The results of modelling for the aortic aneurysms. The values of pressure drops (obtained by CFD), compliances (obtained by the mathematics) and final Diastolic/Systolic pressures of different sections of the aorta with the aneurysms (obtained by electrical system). Please note that the values are approximate.

Abdominal I (Dia = 12.2 mm, L = 63 mm)	ΔP_systole _(pa) (fusiform)	C_aneurysm_(ml/kpa) (fusiform)	ΔP_systole _(pa) (saccular)	C_aneurysm_(ml/kpa) (saccular)	Diastol/Systole Pressure(mmHg) (fusiform)	Diastol/Systole Pressure(mmHg) (Saccular)
20% Aneurysm	70.34	0.212	84.3	0.1985	55–150	55–155
40% Aneurysm	69.9	0.302	79	0.2038	55.2–149.7	55.3–154
50% Aneurysm	69	0.36	76.4	0.22836	56–149.2	55.2–153
70% Aneurysm	65	0.504	74.6	0.28677	56.8–148.7	56.1–152.1
90% Aneurysm	59.5	0.692	74.1	0.3587	58–148	56.8–151

Abdominal II (Dia = 11.7 mm, L = 116 mm)	ΔP_systole _(pa) (fusiform)	C_aneurysm_(ml/kpa) (fusiform)	ΔP_systole _(pa) (saccular)	C_aneurysm_(ml/kpa) (saccular)	Diastol/Systole Pressure(mmHg) (fusiform)	Diastol/Systole Pressure(mmHg) (Saccular)

20% Aneurysm	83.4	0.4785	87.9	0.4454	58–158	57–156
40% Aneurysm	75.73	0.805	85	0.5678	60–155	59–155
50% Aneurysm	74.8	1.0	84.2	0.6438	62–152	60–154
70% Aneurysm	62.4	1.385	83	0.745	64–148	61.5–152
90% Aneurysm	61.5	2.55	82.1	1.0625	70–147	63–150

Abdominal III (Dia = 10.4 mm, L = 10 mm)	ΔP_systole _(pa) (fusiform)	C_aneurysm_(ml/kpa) (fusiform)	ΔP_systole _(pa) (saccular)	C_aneurysm_(ml/kpa) (saccular)	Diastol/Systole Pressure(mmHg) (fusiform)	Diastol/Systole Pressure(mmHg) (Saccular)

20% Aneurysm	70.4	0.225	114	0.03184	55–151	55–163
40% Aneurysm	69.6	0.3099	110	0.0406	54–150	56–161
50% Aneurysm	69.1	0.362	105	0.04603	53.8–149.7	57–160
70% Aneurysm	64	0.489	100.8	0.0592	52–148	59–159.2
90% Aneurysm	58.37	0.65	97.6	0.07605	55–146	60–158.3

Thoracic I (Dia = 20 mm, L = 52 mm)	ΔP_systole _(pa) (fusiform)	C_aneurysm_(ml/kpa) (fusiform)	ΔP_systole _(pa) (saccular)	C_aneurysm_(ml/kpa) (saccular)	Diastol/Systole Pressure(mmHg) (fusiform)	Diastol/Systole Pressure(mmHg) (Saccular)

20% Aneurysm	26.2	0.61475	-	-	53–135	-
40% Aneurysm	23.4	0.846	-	-	54–133	-
50% Aneurysm	21.9	0.9915	-	-	56–132.5	-
70% Aneurysm	21.5	1.347	-	-	56.5–132	-
90% Aneurysm	20.66	1.7984	-	-	59–130	-

Thoracic II (Dia = 13.5 mm, L = 104 mm)	ΔP_systole _(pa) (fusiform)	C_aneurysm_(ml/kpa) (fusiform)	ΔP_systole _(pa) (saccular)	C_aneurysm_(ml/kpa) (saccular)	Diastol/Systole Pressure(mmHg) (fusiform)	Diastol/Systole Pressure (Saccular)

20% Aneurysm	63.7	0.505	-	-	57–149	-
40% Aneurysm	51.05	0.918	-	-	61–147	-
50% Aneurysm	50.75	1.15	-	-	61.8–146.5	-
70% Aneurysm	44.05	1.775	-	-	66–143.5	-
90% Aneurysm	42.2	2.6259	-	-	68–142	-

### 2) The renal artery stenosis

**Figure 9:**
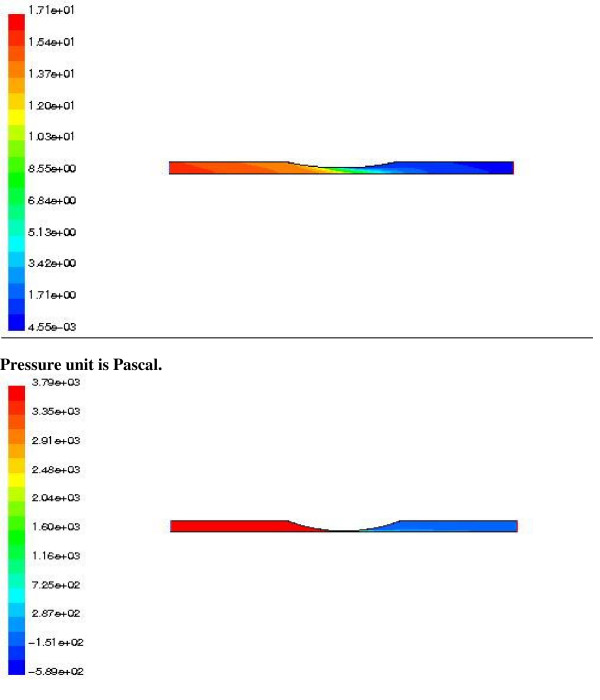
**The pressure contours of the renal artery with 50% (above) and 90% stenosis (under)**. The pressure contour of the renal artery with 50%(above) and 90%(under) narrowing rate of stenosis obtained by CFD method. The high pressure (pascal) regions along the artery sections are at the inlet to the middle portion.

**Figure 10:**
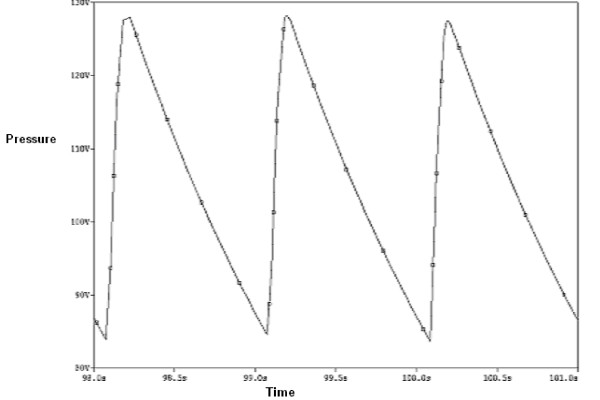
**The electrcal pressure graph of the renal artery with the 50% stenosis**. The pressure waive form of the renal artery with the 50% narrowing rate of stenosis which obtained from the electrical circuit.

Table 3 shows the results of the modeling including the pressure drops, at peak systole, and the compliances of the renal artery with different rate of the stenosis. Figure 9 shows the variations of pressure along the renal artery length, with 50% and 90% stenosis, at peak systole. The high pressure regions along the artery sections are at the inlet to the middle portion. The effects of the renal stenosis were also studied using the electronic system. The diastole/systole pressure graph of the renal artery, with 50% stenosis, is presented in Figure 10. The variation of pressure waveform is between 85–127 mmHg (volt) during the cardiac cycle.

**Table 3:** The results of modelling for the renal stenosis. The values of pressure drops (obtained by CFD), compliances (obtained by the mathematics) and final Diastolic/Systolic pressures of the renal with stenosis (obtained by electrical system). Please note that the values are approximate.

Renal (Dia = 2.6 mm, L = 32 mm)	ΔP(Pa) Stenosis	C(ml/Kpa) Stenosis	Diastol/Systole Pressure(mmHg)
20% Stenosis	7.46	0.01512	82–121
50% Stenosis	17.1	0.0236	85–127
70% Stenosis	75	0.116	97–148
90% Stenosis	3790	0.136	145–210

## Discussion

We have modeled the aorta aneurysms and the renal stenosis by means of the computational fluid dynamics and mathematical methods. The results were shown for different pressure graphs in the cardiovascular electronic system. The aortic aneurysm and the renal stenosis cause hypertension which is defined as increasing of blood pulse pressure.

### 1) The aorta artery aneurysms

The aorta aneurysm expansion rate is a function not only of initial size, but also of blood pressure. This indicates that increasing pulse pressure is associated with the aneurysm expansion rate [5]. Our resulting pressure drops in the present study agree with the results in Schurink's paper [3]. Our results, Table 2, show that pulse pressure increases with the rate of both aortic aneurysms including saccular and fusiform. Furthermore, clinical investigations demonstrate that 70–90% of patients with aortic aneurysms have high blood pressure [4] and supports the clinical importance of blood pressure control in reducing the risk of rupturing in the aortic aneurysms. On the other hand, the most effective factor which controls the hypertension of the aortic aneurysms is compliance [3]. Furthermore, alerted elastic properties and a reduced concentration of aortic elastic both have been associated with the aortic aneurysms [3]. In the present study, the compliance of the aorta artery increases with the rate of the aneurysms. The clinical investigations [4] imply that an increase in the compliance at the maximum aneurysm diameter was associated with rupture or need for surgery. We have shown that the higher aneurysm rate causes more hypertension and increasing of the pulse pressure in the cardiovascular system. The clinical data reported the pulse pressure of 52.5–120 mmHg for the aortic aneurysms depending on the rate of the aneurysms [15]. Our results were compared with these data and were in good agreement with them. We have found the average increase of 85 mmHg (volt) in the pulse pressure of the abdominal I aortic aneurysms. The values are 82 mmHg for the abdominal II, 77 mmHg for the thoracic I and 85 mmHg for the thoracic II.

### 2) The renal artery stenosis

The previous studies [6-8] found a positive correlation between blood pulse pressure and the rate of renal stenosis and noted that the hypertension due to the stenosis depends to many genetic factors such as fibrillin. The investigations indicate that increasing pulse pressure is associated with the stenosis expansion rate which leads to hypertension [6]. The other clinical data [9] reported the renal systolic pressure to be 124–166 mmHg and the diastolic to be 84–108 mmHg due to the rate of the stenosis. Our modelling results, Table 3, report the significant increase in the pulse pressure due to the stenosis narrowing rate. These values have the average of 102 mmHg (volt) for diastolic and 152 mmHg (volt) for systolic. This implies that the modelling results are in a good agreement with the clinical data.

## Conclusion

In summary, our results indicate that expansion rate of the aortic aneurysms as well as the narrowing rate of the renal stenosis both are directly associated with hypertension, supporting the clinical importance of blood pressure control. We hereby stress that our model is an ideal and general model of the cardiovascular system. This electrical model proves useful for studying the pathogeneses of the cardiovascular system as discussed above.
